# Middle Hepatic Vein-Guided Cranial and Hilar Approach Using a Modified Two-Surgeon Technique in Laparoscopic Extended Left Medial Sectionectomy: A Technical Case Report With Videos

**DOI:** 10.7759/cureus.37865

**Published:** 2023-04-20

**Authors:** Shigetoshi Naito, Takahisa Fujikawa, Masatoshi Kajiwara, Suguru Hasegawa

**Affiliations:** 1 Gastroenterological Surgery, Fukuoka University Hospital, Fukuoka, JPN; 2 Surgery, Kokura Memorial Hospital, Kitakyushu, JPN

**Keywords:** two-surgeon technique, anatomical landmark, hepatic vein, anatomical resection, laparoscopic liver resection

## Abstract

Anatomical liver resection and liver resection close to major blood vessels are quite challenging and require a high level of expertise. In addition, anatomical hepatectomy requires extensive knowledge of the positions of blood vessels and techniques for hemostasis because the resection surface is extensive and operations around blood vessels are required. A hepatic vein-guided cranial and hilar approach using a modified "two-surgeon technique" is effective in resolving these problems. Herein, we present a middle hepatic vein (MHV)-guided cranial and hilar approach using a modified two-surgeon technique in laparoscopic extended left medial sectionectomy to resolve these problems. This procedure is feasible and effective.

## Introduction

We believe that laparoscopic liver resection (LLR) may reduce invasiveness and facilitate postoperative functional recovery [1,2]. LLR has been shown to be useful not only for HCC but also for metastatic liver tumors [3,4]. Therefore, the use of laparoscopic surgery to replace traditional open liver surgery (OLR) is increasing worldwide [5,6]. However, LLR is more difficult than ordinary laparotomies, and more time is required to learn the procedure. In a report by Ban et al. [7], the degree of difficulty of laparoscopic liver resection was scored based on the following five items: tumor location, resection method, tumor size, relationship with major vessels, and liver function.

One of the most important tasks during liver resection is minimizing intraoperative surgical blood loss; hence, in this report, we present the improved outcomes of the "two-surgeon technique" [8]. The two-surgeon technique was first utilized for open liver resections and was found to improve several outcomes [9,10]. We applied this approach to LLR [8]. Using this technique, rapid laparoscopic liver parenchymal transection comparable to open liver resection can be achieved. With this technique, rapid hemostasis is possible owing to a clear division of roles (e.g., liver resection can be managed by the main surgeon and hemostasis by the assistant surgeon). The concepts of "role sharing" and "mode switching" can be used to accomplish satisfactory liver parenchymal transection and hemostasis. The primary surgeon dissects the hepatic parenchyma, whereas the secondary surgeon employs saline-linked electrocautery (SLiC) to focus on hemostasis. However, in the event of bleeding from the deep parenchymal fissure or a major hepatic vein, the secondary surgeon switches roles ("role sharing") with the primary surgeon. Subsequently, the secondary surgeon returns to the role of SLiC.

Herein, we report on laparoscopic extended left medial sectionectomy that includes the ventral part of S8 and S5 using the "two-surgeon technique" for S8 rectal liver metastasis close to the middle hepatic vein (MHV).

## Technical report

A 46-year-old female patient was diagnosed with rectosigmoid cancer and synchronous liver metastases at a nearby hospital and underwent laparoscopic colon surgery. Postoperative XELOX plus bevacizumab therapy was initiated but discontinued because of myelosuppression. The patient was referred to our department because she requested laparoscopic resection of the liver metastases during drug withdrawal.

**Figure 1:**
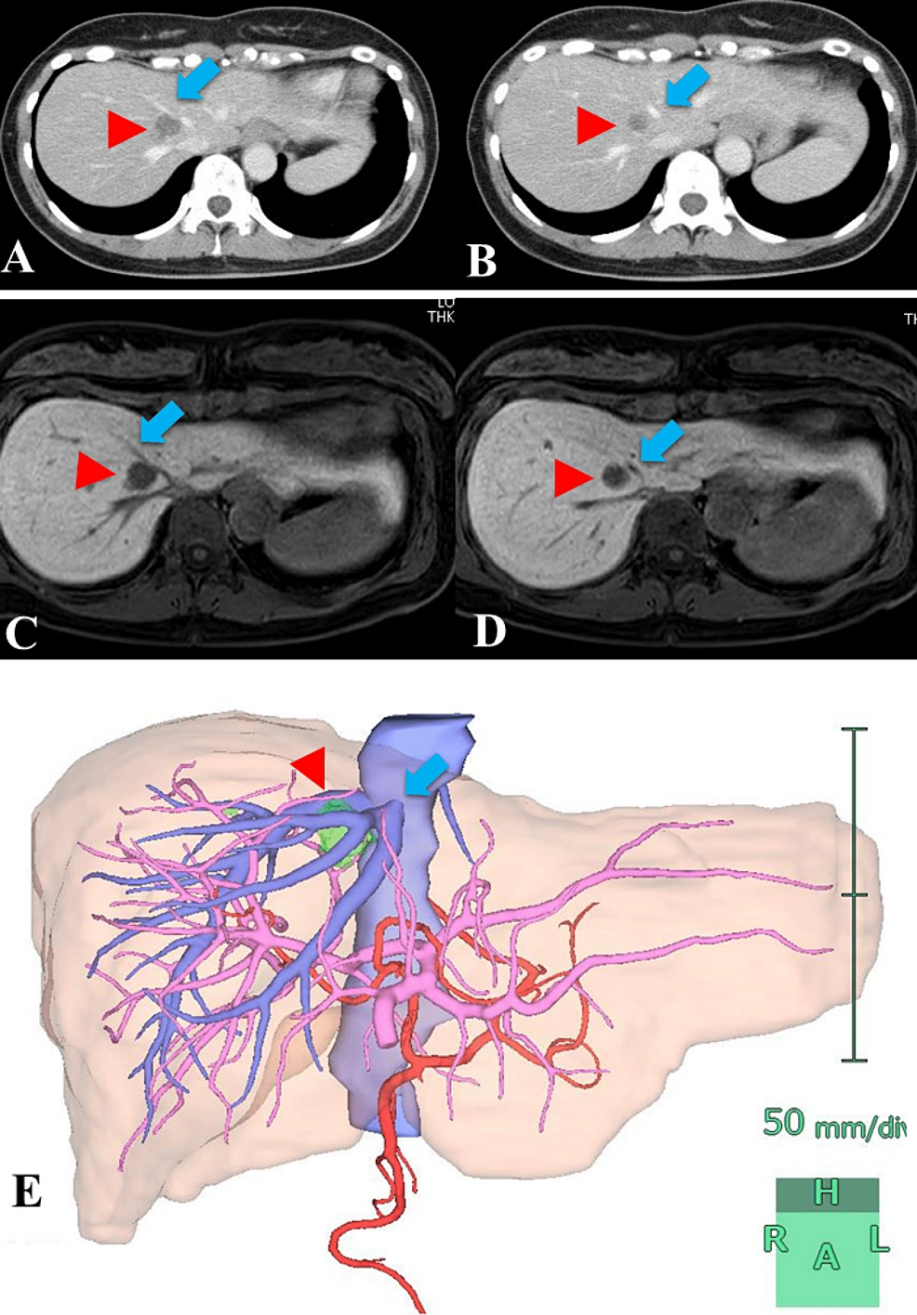
Preoperative image in the current case (A) and (B) show contrast-enhanced CT findings. (C) and (D) show the Gd-EOB-DTPA MRI study. (E) shows the results of the 3D construction. (Red arrowheads indicate the tumor. Blue arrows indicate the middle hepatic vein.) The tumor with a maximum diameter of 20 mm in S8 was small, but the lesion was located at a site sandwiched between the RHV and the root of the MHV. CT: computed tomography, Gd-EOB-DTPA: gadolinium ethoxybenzyl-diethylenetriaminepentaacetic acid, MRI: magnetic resonance imaging, RHV: right hepatic vein, MHV: middle hepatic vein

Preoperative imaging confirmed the presence of a tumor with a maximum diameter of 20 mm in S8 (Figure 1). In addition, S2 and S3 each had liver metastases of 10 mm in size. Although the tumor in S8 was small, the lesion was localized at the site between the right hepatic vein (RHV) and the root of the MHV. Regarding the appropriate approach, we judged that resection of the MHV was unavoidable and planned an extended left medial sectionectomy with MHV resection. In other words, we planned to resect the left medial segment including the ventral part of S8 and S5, which were drained by the middle hepatic vein (MHV). It was also necessary to enter the inferior vena cava (IVC) anteriorly to provide a dorsal margin for the tumor. An approach from S8 might require anterior segment resection; however, the small size of S4 made an approach from here reasonable in terms of preserving the hepatic reserve. Prior exposure of the main hepatic vein is usually performed in anatomical hepatectomy; however, in this case, the MHV prior approach was used to understand the localization of the tumor.

A liver parenchymal transection-first approach guided by the MHV

**Figure 2:**
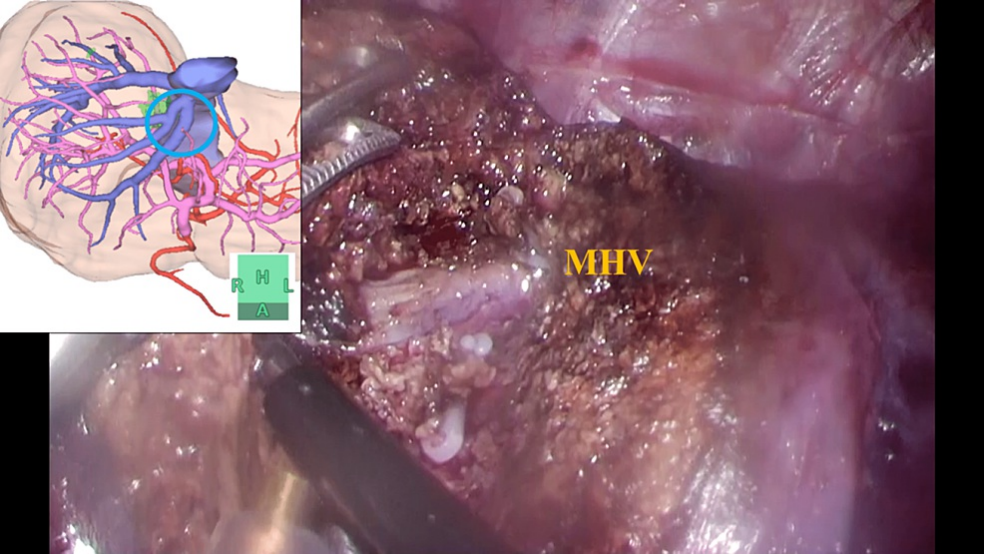
Exposing the MHV Hepatic parenchymal transection just above the root of the MHV and the MHV was exposed to the operative field. MHV: middle hepatic vein

The ports were concentrated around the tumor. We separated the round and falciform ligaments of the liver and performed the Pringle maneuver (extracorporeal tourniquet method) after cholecystectomy. The RHV, MHV, and tumor localization were confirmed using intraoperative ultrasonography (US). A line that exposes the root of the MHV was set as the scheduled separation line. Subsequently, the planned resection line of S4 was marked and connected to the marking of the MHV root, followed by hepatic parenchymal transection just above the root of the MHV. At that time, the operative field was maintained dry by carefully switching to the hemostatic mode using the modified two-surgeon technique [8]. Subsequently, the MHV was exposed to the operative field (Figure 2, Video 1).

**Video 1:** Exposing the MHV MHV: middle hepatic vein

Liver parenchymal dissection of the left dissection line from the caudal side

**Figure 3:**
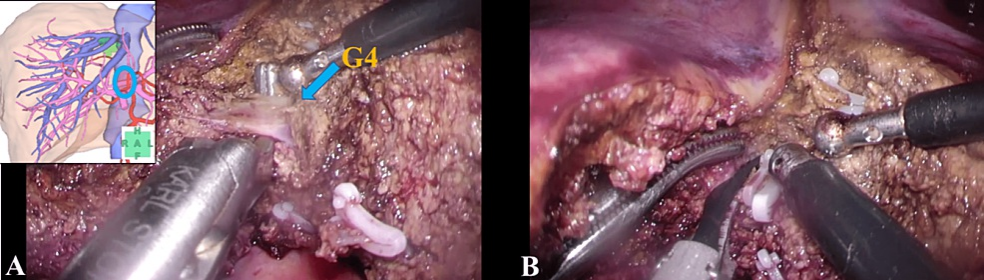
Exposing and resecting the G4 (A) shows the exposed root of the G4. (B) shows that the root of the G4 was resected by laparoscopic coagulating shears after double clipping only on the central side.

Once the MHV root was well exposed, dissection of S4 commenced from the caudal side. In the current case, liver injury was caused by chemotherapy, and the liver parenchyma was soft and fragile. In such cases, hemostasis with fine mode switching using the two-surgeon technique is particularly effective. While being conscious of mode switching, the roles of resection and hemostasis were thoroughly shared, and liver resection was quickly performed. The root of the G4 was exposed, and double clipping only on the central side was performed. The resection side could be handled only by sealing in the laparoscopic coagulating shears (Figure 3, Video 2).

**Video 2:** Exposing and resecting the G4

Exposing and dividing the G5 and widening the cut surface on the liver caudal side toward the root of the MHV

**Figure 4:**
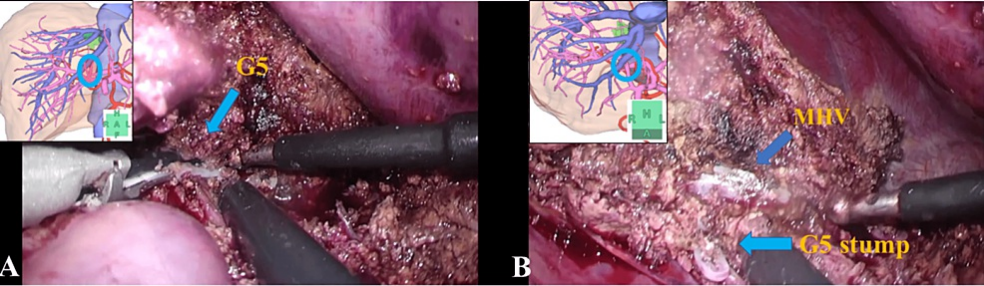
Exposing and resecting the G5 (A) shows the exposed root of the G5. (B) shows that the periphery of the MHV was confirmed cranially after resecting the G5. MHV: middle hepatic vein

Dorsal dissection was performed to further widen the caudal view. We exposed and divided the G5 from the anterior segment of the Glissonean pedicle and clipped it. Subsequently, the periphery of the MHV was confirmed cranially. In MHV resection, the dorsal detachment of the MHV was widened to form the resection line. Subsequently, parenchymal transection was performed on the right and dorsal sides in the same way along the demarcation line, which was determined by dividing the G5 branch. We then connected the dissection line to the previously exposed MHV and widened the window. Thus, it was easier to approach the MHV root (Figure 4, Video 3).

**Video 3:** Exposing and resecting the G5

Exposing and resecting the MHV root

**Figure 5:**
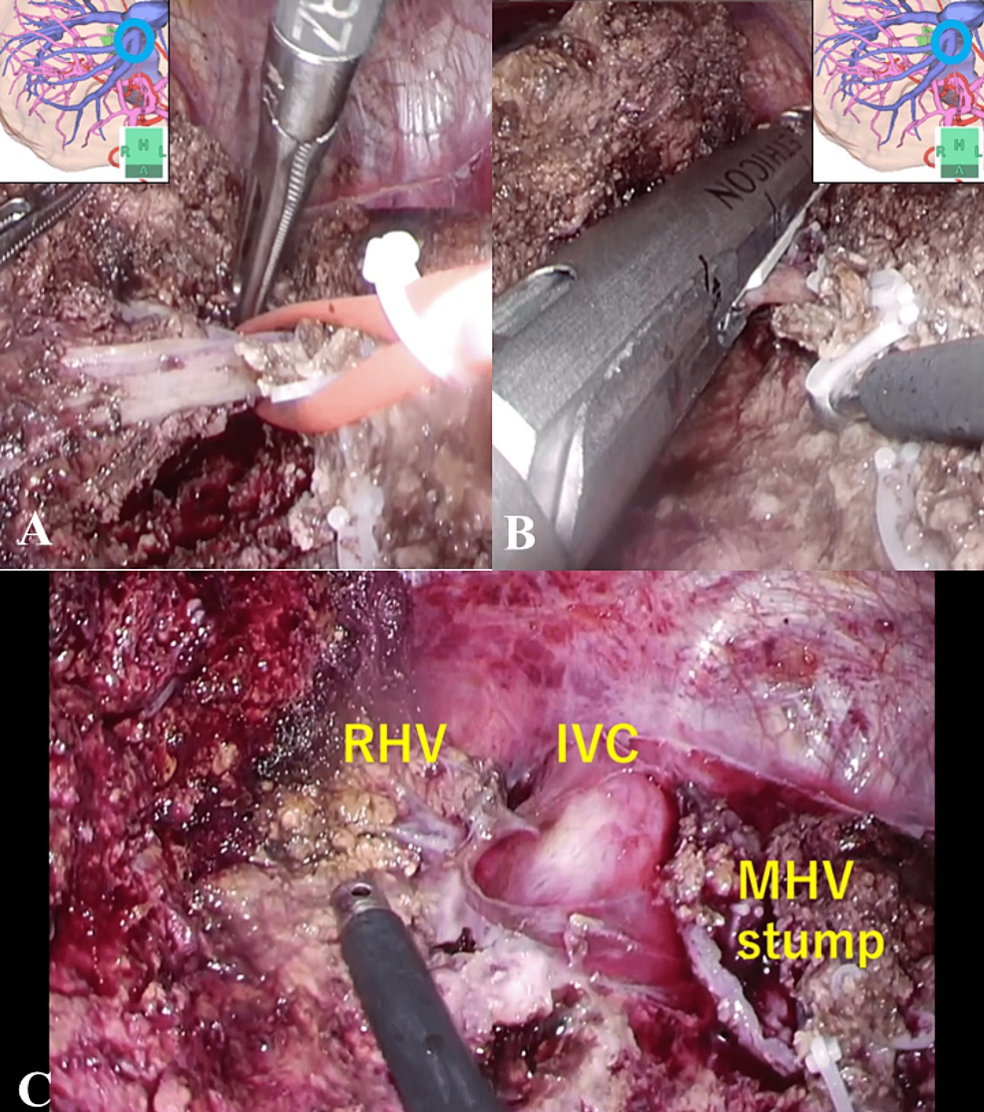
Exposing and resecting the MHV root (A) shows that the root of MHV is sufficiently exposed. (B) shows that the MHV was resected by an auto-suture device. (C) shows that the MHV stump and the IVC and RHV were exposed after the excision of the specimen. MHV: middle hepatic vein, RHV: right hepatic vein, IVC: inferior vena cava

After carefully peeling off the area around the base of the MHV while switching modes, the vessel was exposed. Furthermore, the cranial side of the liver parenchyma was sufficiently crushed to create space for inserting an auto-suture device. The IVC and RHV were exposed dorsally immediately after the MHV amputation. The dissection of the liver parenchyma was gently advanced to the right, forming a line that wrapped around the dorsal side of the tumor along the anterior surface of the RHV. Finally, the liver parenchyma was transected from the caudal to the cranial side. As large vessels were rarely encountered in this region, we dissected them to connect the dissection line from the right and left sides of the tumor. Hence, hemostasis was appropriately performed by mode switching using the two-surgeon technique to quickly proceed while managing hemorrhage. After exposing and dissecting the G8vent branch and the peripheral branch of V8, the dissection of the liver parenchyma was advanced until the dissection was completed (Figure 5, Video 4). Tumors were confirmed in S3 and S2, and additional partial excisions were performed.

**Video 4:** Exposing and resecting the MHV root MHV: middle hepatic vein

The Pringle maneuver was performed only if there was excessive bleeding, and the blood flow was intermittently interrupted for 15 minutes. Finally, the blood flow was interrupted three times (45 minutes) before the resection was completed. The total operation time was 497 minutes, and the total blood loss was 370 g. The postoperative course was uneventful, and the patient was discharged after seven days postoperatively.

## Discussion

The middle hepatic vein-guided cranial and hilar approach was used to successfully perform an extended left medial sectionectomy. In addition, a difficult anatomical resection, such as one that is close to the main vessels, was completed without major incidents using the modified "two-surgeon technique."

Surgical resection is considered the primary treatment option for liver metastases in patients with colorectal cancer. Local ablation therapy is less recommended than hepatic resection for patients with colorectal liver metastases [11]. The invasiveness of surgery is also a concern; however, the patient in this case was young and poorly tolerated chemotherapy, and more effective treatment was considered necessary. Previous studies have reported that intraoperative blood loss, morbidity rate, and postoperative hospital stay are significantly lower in patients who underwent LLR. Furthermore, there was no difference in long-term survival. Therefore, LLR is considered a minimally invasive procedure.

One of the technical challenges of LLR is the difficulty in obtaining an accurate stereoscopic space inside the liver [12]. Therefore, we believe that the resection line is often different from what was initially planned. However, if the target vessel can be identified first, it is an effective reference landmark for the line depth of resection. The main vessels within the liver are the Glisson and hepatic veins, each with its own antecedent approach. In the Glissonean approach, the line of dissection is determined by confirming the ischemic zone. However, during resection, it is sometimes difficult to determine the ischemic area inside the liver parenchyma and confirm the position of the resection line in real time without a target, even under US guidance. It is also difficult to locate the tip of the hepatic vein within the liver. Therefore, anatomical liver resection may be easier with exposed hepatic veins than with the Glissonean pedicle approach. However, because small blood vessels join the main veins, there is a risk of bleeding due to injury, and techniques to prevent bleeding are required. Honda et al. [13] reported that a non-traumatic point is to move the cavitron ultrasonic surgical aspirator proximally and distally to avoid splitting the venous confluence. The location of the tumor in this case was particularly close to the dorsal side of the MHV, and it was deep. Understanding the resection line is important for the complete resection of the tumor, and the hepatic vein-first approach is very effective. Furthermore, the goal can be clearly determined even when the remaining part of the body is separated from the caudal side; therefore, separation can be confidently performed. In another report of left medial transection, Glisson's pretreatment was performed, and an intracranial approach along the ischemic zone was used [14]. The concept is similar; however, in this case, the root of the MHV must be treated particularly well. Exposing the MHV before dividing into the G4, combined with caudal dissection, would have clarified directionality and determined a deeper and more effective line of dissection.

Anatomical hepatectomy requires techniques for hemostasis because the resection surface is extensive and operations around blood vessels are required. We have been making full use of the modified "two-surgeon technique" as a method for efficiently achieving hemostasis. By thoroughly sharing roles, satisfactory liver parenchymal transection can be achieved. The two-surgeon technique is composed of the "transection mode" and the "hemostatic mode." These modes are switched rigidly according to the surgical field condition to ensure ideal "role sharing." We previously reported that this technique results in lower transfusion rates and blood loss compared to the rates in hybrid liver resection [8]. Improving the efficiency of hemostasis leads to a better field of view and more stable dissection, which is advantageous for surgical safety, even in cases of difficult liver resections.

## Conclusions

The hepatic vein-guided cranial and hilar approach for difficult anatomical hepatectomy helps determine the resection line. Furthermore, rapid parenchymal transection through the use of the two-surgeon technique is effective for LLR. The modified two-surgeon technique can be used to control bleeding for LLR with a large resection area.
